# Effects of enalapril and paricalcitol treatment on diabetic nephropathy and renal expressions of TNF-α, p53, caspase-3 and Bcl-2 in STZ-induced diabetic rats

**DOI:** 10.1371/journal.pone.0214349

**Published:** 2019-09-17

**Authors:** Osama M. Ahmed, Tarek M. Ali, Mohamed A. Abdel Gaid, Ahmed A. Elberry

**Affiliations:** 1 Experimental Obesity and Diabetes Research Lab, Physiology Division, Zoology Department, Faculty of Science, Beni-Suef University, Beni-Suef, Egypt; 2 Department of Clinical Laboratories, College of Applied Medical Sciences, Taif University, Taif, Saudi Arabia; 3 Department of Physiology, Faculty of Medicine, Beni-Suef University, Beni-Suef, Egypt; 4 Clinical Pharmacology Department, Faculty of Medicine, Beni-Suef University, Beni-suef, Egypt; National Institutes of Health, UNITED STATES

## Abstract

This study aimed to assess the renopreventive effect of enalapril and/or paricalcitol on streptozotocin (STZ) diabetes-induced nephropathy and to elucidate their mechanisms of action through investigation of the effects on renal oxidative stress, antioxidant defense system and expressions of TNF-α, p53, caspase-3, and Bcl-2. Diabetes mellitus was induced in fasting male Wistar rats by single intraperitoneal injection of STZ (45 mg /kg b.w.) dissolved in citrate buffer (pH 4.5). Ten days after STZ injection, the diabetic rats were treated with enalapril (25 mg/l of drinking water) and/or paricalcitol (8 μg/kg b.w. *per os*) dissolved in 5% DMSO daily for 4 weeks. The obtained data revealed that the treatment of diabetic Wistar rats with enalapril and/or paricalcitol led to significant decreases in the elevated serum urea, uric acid, creatinine, sodium and potassium levels; thereby reflecting the improvement of the impaired kidney function. The deteriorated kidney lipid peroxidation, GSH content and GST and catalase activities in diabetic rats were significantly ameliorated as a result of treatment with enalapril and/or paricalcitol. The elevated fasting and post-prandial serum glucose levels and the lowered serum insulin and C-peptide levels were also improved. The treatment with enalapril and paricalcitol in combination was the most potent in decreasing the elevated serum glucose levels. Moreover, the treatment of diabetic rats successfully prevented the diabetes-induced histopathological deleterious changes of kidney and islets of Langerhans of pancreas. In association, the immunohistochemically detected pro-inflammatory cytokine, TNF-α, and apoptotic mediators, p53 and caspase-3, were remarkably decreased in kidney of diabetic rats as a result of treatment while the expression of anti-apoptotic protein Bcl-2 was increased. Based on these findings, it can be concluded that enalapril and paricalcitol alone or in combination can prevent STZ diabetes-induced nephropathy through amelioration of the glycemic state and antioxidant defense system together with the suppression of oxidative stress, inflammation and apoptosis. However, the treatment of diabetic rats with enalapril and paricalcitol in combination has no further significant improvement effects on renal function and damage when compared with enalapril or paclitaxel treated diabetic groups.

## Introduction

Diabetic nephropathy (DN), a complicated microvascular disease associated with diabetes mellitus, is a significant cause of chronic kidney disease (CKD) [1]. In a type 1 diabetes mellitus (T1DM) population with a mean age of 44 years and duration of diabetes of 17 years, up to 21.0% of patients developed CKD [2]. Similarly, DN occurs in 20% to 40% of all cases of type 2 diabetes mellitus (T2DM) [3]. Approximately 14.5% mortality was recorded among people with DM (aged 20 to 79 years) [4]. This increased risk may be essentially driven by kidney disease which may be correlated with the presence of atherosclerosis or diabetes-associated glomerular damage [5] probably with the incidence of severe interstitial inflammation [6].

The prevention of activated renin-angiotensin-aldosterone system (RAAS) in diabetic kidney has a vital role in treatment of DN [7]. Drugs that block RAAS pathway are effective in reducing nephropathy progression in diabetic patients and delaying cardiovascular and renal morbidity and mortality [8].

Enalapril is one of angiotensin converting enzyme inhibitors (ACEIs), a class of antihypertensive medications, that have been shown to be of greater benefit in preventing RAAS, reducing DN, abating progression of renal failure, restoring glomerular hyperperfusion and hyperfiltration, improving glomerular barrier function, and reducing the non-hemodynamic effects of angiotensin II and aldosterone [9,10]. Moreover, Vermes *et al*. [11] reported that enalapril markedly reduces the risk of developing diabetes in patients with left ventricular systolic dysfunction by elevation of insulin sensitivity, skeletal muscle glucose uptake, and pancreatic blood flow. In the same way, Shamiss *et al*. stated that enalapril improves some of the metabolic parameters including insulin sensitivity in hypertensive diabetic patients [12].

Paricalcitol (19-nor-1, 25-dihydroxyvitamin D2), an active non-hypercalcemic selective vitamin D analogue and an activator of vitamin D receptor (VDR), was reported to inhibit activated RAAS by decreasing renin, renin receptor, angiotensinogen and angiotensin type 1 receptor [13]. It was reported that renin inhibition could benefit patients with metabolic syndrome independently of blood pressure reduction [14]. Studies in experimental nephropathy animal models have demonstrated that paricalcitol improves glomerular degeneration and tubular damage [15]. Clinically, Aperis *et al*. [16] demonstrated an average 32.9% reduction of proteinuria in 19 patients treated with 1–2 μg daily paricalcitol with 74% of patients respond, especially in DN. Izquierdo *et al*. [17] found that paricalcitol has antioxidant effects as it decreases serum malondialdhyde (MDA) levels and increases serum reduced glutathione (GSH) and thioredoxin levels, as well as superoxide dismutase (SOD) and catalase (CAT) activities in hemodialysis patients.

The combined use of enalapril and paricalcitol, both alter RAAS [10,18], was investigated by various authors who assessed their improvement effects on blood pressure, urinary protein excretion, renal function and kidney histological architecture in uremic rats [19] as well as on the heart function and cardiac antioxidant defense in diabetic rats [20]. In addition, Husain *et al*. [21] reported that paricalcitol and enalapril alone or in combination ameliorated the blood pressure and renal lipid peroxidation (LPO), antioxidant defense system and inflammatory proteins in ApoE-deficient atherosclerotic mice; the effect paricalcitol and enalapril in combination was the most potent on renal oxidative stress and antioxidant defense system. Sagar *et al*. [22] stated that paricalcitol potentiated the anti-hypertensive action of enalapril in experimental PKD but did not reduce renal cyst growth. The mechanisms of action and the roles of oxidative stress, inflammation and apoptosis and their possible links as a result of treatment of DN with enalapril and paricalcitol in combination are not fully elucidated and needs further investigations.

Therefore, this study was conducted to assess the renopreventive effect of enalapril and paricalcitol on streptozotocin (STZ)-induced nephropathy and try to elucidate the roles of renal oxidative stress, antioxidant defense system, inflammation and apoptosis through scrutinizing the effects on the renal LPO and antioxidant defense markers as well as the renal expressions of tumor necrosis factor-α (TNF-α), caspase-3, B cell lymphoma-2 (Bcl-2) and protein 53 (p53).

## Materials and methods

### Experimental animals

Male albino rats of Wistar strain weighing about 100–130 grams were used in this study. After two weeks of adaptation period, the animals were housed in clean polypropylene cages and maintained in an air-conditioned animal house at temperature of 20–25°C with natural alternating light and dark cycles. The animals were supplemented with standard pellet diets and water *ad libitum*. All experimental procedures were approved by Experimental Animal Ethics Committee of Faculty of Science, Beni-Suef University, Beni-Suef, Egypt (Ethical Approval Number: BSU/FS/2016/11). All efforts were done to minimize animal pain and suffering.

### Induction of diabetes mellitus

Diabetes mellitus was induced in male Wistar rats by a single intraperitoneal injection of STZ (Sigma, St. Louis, MO, USA) at dose level of 45 mg /kg body weight (b.w.) dissolved in citrate buffer (pH 4.5) [23]. Ten days after STZ injection, animals were deprived of food and water overnight (10–12 h) and blood samples were withdrawn from lateral tail vein at fasting state and 2 hours (hr) of oral glucose loading (3 g/kg b.w.). Serum was aspirated after centrifugation of coagulated blood of each rat at 3000 round per minute (r.p.m.) for 15 minutes and serum glucose concentration was determined. Rats of a 2-hr serum glucose level from 180 to 300 mg/dl were considered as mild diabetic while those outside this range were excluded as indicated in our previous publication [23].

### Animal grouping

After induction of diabetes mellitus, the rats were allocated into the five groups (6 rats for each group): Group 1 (Normal group) was intraperitoneally injected with the equivalent volume of the vehicle (5% dimethyl sulphoxide [DMSO]) daily for 4 weeks. Group 2 (Diabetic control group) was composed of diabetic rats that were intraperitoneally injected with the equivalent volume of the vehicle (5% DMSO) daily for 4 weeks. Group 3 (Diabetic group treated with enalapril) included diabetic rats that were treated with enalapril in drinking water at concentration 25 mg enalapril/l of drinking water for 4 weeks; this group was intraperitoneally injected with the equivalent volume of 5% DMSO. Group 4 (Diabetic group treated with paricalcitol) consisted of diabetic rats that were intraperitoneally injected with paricalcitol at dose level of 8 μg/kg b.w. dissolved in 5% DMSO daily for 4 weeks. Group 5 ((Diabetic group treated with enalapril and paricalcitol) was composed of diabetic rats that were treated with enalapril in drinking water (25 mg enalapril/l) and were also intraperitoneally injected with pariclacitol at dose level of 8 μg/kg b.w. dissolved in 5% DMSO daily for 4 weeks.

### Blood and tissue sampling

At the day before the sacrifice at the end of the experiment, blood samples were obtained from lateral tail vein at fasting state and after two hours of oral glucose loading (3 g/kg b.w. by oral gavage) to measure serum glucose levels. At the following day, animals were sacrificed under anaesthesia. Blood sampling, separation of serum, kidney sampling, kidney homogenization and kidney fixation were performed as described in detail in our previous publication [24].

### Biochemical investigations

Serum urea level was determined using kits obtained from BIOMED Diagnostic (EGY-CHEM for Lab Technology) Bader city, Cairo, Egypt. Serum uric acid was determined using reagent kits purchased from Spinreact, S.A.U. (SPAIN). Serum creatinine was detected using reagent kits obtained from Diamond Diagnostic Chemical Company (Egypt). Measurements of serum sodium and potassium ions were carried out using reagent kits purchased from Spectrum Company for Biotechnology, Obour City, Cairo, Egypt. Serum glucose level was determined by using commercial diagnostic kit obtained from Randox Laboratories, UK. Serum insulin and C-peptide levels were assayed using enzyme-linked immunosorbent assay kits purchased from Linco Research, St. Charles, MO, USA according to manufacturer’s instruction. Kidney GSH level, lipid peroxidation (LPO) represented by MDA level, glutathione-S-transferase (GST) activity, and catalase (CAT) activity were determined according to the chemical methods of Beutler *et al*. [25], Preuss *et al*. [26], Mannervik and Gutenberg [27] and Cohen *et al*. [28] respectively.

### Histological and immunohistochemal investigations

Kidneys, fixed in neutral buffered formalin, were transferred to the National Cancer Institute, Cairo University, Egypt for blocking in wax, sectioning and staining with hematoxylin and eosin (H & E) according to the method of Banchroft *et al*. [29]. Then, the prepared stained sections were examined to detect the histological changes.

Immunohistochemical techniques for TNF-α, p53, caspase-3 and Bcl-2 by using 3 μm thickness liver sections mounted on positive glass slides according to the methods of Hussein and Ahmed [30] respectively. Examination and analysis of labeling was performed using ImageJ software (1.51d) to measure the integrated intensities and area percent for positive immunohistochemical reactions in a standard measuring frame of the captured images.

### Statistical analysis

The data were analysed using the one-way analysis of variance (ANOVA) (PC-STAT, 1985, University of Georgia, USA) [31] followed by LSD analysis to compare various groups with each other. Results were expressed as mean ± standard error (SE). F-probability obtained from one-way ANOVA, expresses the effect between groups.

## Results

Serum urea, uric acid and creatinine levels were significantly (p<0.01; LSD) elevated in diabetic control group while these elevated levels were remarkably decreased in diabetic treated groups with F-probabilities P<0.001, P<0.05 and P<0.01 respectively (Table 1). The treatment with enalapril produced the most potent effects in decreasing the elevated serum urea, uric acids and creatinine levels recording percentage changes of -49.36, -39.72 and -41.50% respectively. The diabetic rats treated with enalapril and paricalcitol in combination exhibited no further significant effects (P>0.05) on serum urea, uric acid and creatinine levels when compared with the diabetic groups singly treated with either enalapril or paricalcitol (Table 1). Both serum sodium and potassium levels were significantly increased (p<0.01; LSD) in diabetic control rats as compared with normal control; the recorded percentage changes were 3.59 and 22.04% respectively. The treatment of diabetic rats with enalapril, paricalcitol and their combination significantly normalized the elevated serum sodium and potassium levels when compared with diabetic control with F-probabilities P<0.001 and P<0.05 respectively. The effect of the treatment with enalapril and paricalcitol in combination on serum sodium and potassium levels was not significant (P>0.05) as compared to the treatment with either enalapril or paricalcitol (Table 2). The elevated kidney LPO represented by MDA level was normalized by the treatment with enalapril, paricalcitol and their combination recording percentage changes of -40.11, -44.60 and -36.57 respectively. However, the treatment with enalapril and paricalcitol in combination produced no further significant effect (P>0.05) on kidney LPO when compared with the diabetic groups treated singly with enalapril or paricalcitol (Table 3). The lowered kidney GSH content as well as GST and catalase activities in diabetic rats were significantly increased as a result of treatment with enalapril, paricalcitol and their combination with F-probabilities of P<0.001, P<0.01 and P<0.001 respectively (Tables 3 and 4). While the effect of co-treatment with enalapril and paricalcitol was the most potent on GSH content (37.49%), paricalcitol appeared to be the most effective on GST activity (43.64%) and enalapril seemed to be the most effective on catalase activity (40.70%) (Tables 3 and 4). In spite of these changes, the effect of treatment with the combination of enalapril and paricalcitol on kidney GSH content was not significant (P>0.05) as compared with the treatment with enalapril alone (Table 3). Moreover, the diabetic rats treated with enalapril and paricalcitol in combination showed no significant effects (P>0.05) on renal GST and catalase activities as compared to the diabetic groups treated with either enalapril or paricalcitol (Table 4).

**Table 1:** Effects of enalapril and paricalcitol on serum urea, uric acid and creatinine levels in diabetic rats.

	Urea (mg/dl)	% change	Uric acid (mg/dl)	% change	Creatinine (mg/dl)	% change
Normal	29.17 ± 2.29^d^	-	1.31 ± 0.132^b^	-	0.64 ± 0.004^b^	-
Diabetic control	83.52 ± 9.79^a^	186.32	2.19 ± 0.295^a^	67.17	1.06 ± 0.138^a^	63.07
Diabetic treated with Enalapril	42.29 ± 1.54^cd^	-49.36	1.32 ± 0.181^b^	-39.72	0.62 ± 0.003^b^	-41.50
Diabetic treated with Paricalcitol	65.56 ± 5.15^b^	-21.50	1.52 ± 0.140^b^	-30.59	0.78 ± 0.082^b^	-26.41
Diabetic treated with Enalapril and Paricalcitol	55.67 ± 5.17^bc^	-33.34	1.74 ± 0.230^ab^	-20.54	0.70 ± 0.074^b^	-3369
F-probability	P<0.001		P<0.05		P<0.01	
LSD at 5% level	16.322		0.598		0.240	
LSD at 1% level	22.082		0.809		0.325	

- Data are expressed as mean ± SE. Number of detected samples in each group is six.

- Means, which share the same superscript symbol(s) are not significantly different.

- Percentage changes were calculated by comparing diabetic control group with normal control group and diabetic treated groups with diabetic control group.

**Table 2:** Effects of enalapril and paricalcitol on serum sodium and potassium levels in diabetic rats.

	Sodium (mg/dl)	% change	Potassium (mg/dl)	% change
Normal	149.19 ± 0.38^c^	-	4.90 ± 0.21^b^	-
Diabetic control	154.55 ± 0.84^a^	3.59	5.98 ± 0.26^a^	22.04
Diabetic treated with Enalapril	150.27 ± 0.12^bc^	-2.76	5.16 ± 0.04^b^	-13.71
Diabetic treated with Paricalcitol	151.84 ± 0.35^b^	-1.75	5.23 ± 0.35^b^	-12.54
Diabetic treated with Enalapril and Paricalcitol	151.59 ± 0.74^b^	-1.91	4.83 ± 0.15^b^	-19.23
F-probability	P<0.001		P<0.05	
LSD at 5% level	1.627		0.668	
LSD at 1% level	2.202		0.904	

- Data are expressed as mean ± SE. Number of detected samples in each group is six.

- Means, which share the same superscript symbol(s) are not significantly different.

- Percentage changes were calculated by comparing diabetic control group with normal control group and diabetic treated groups with diabetic control group.

**Table 3:** Effects of enalapril and paricalcitol on kidney GSH content and LPO in diabetic rats.

	LPO (nmole MDA/100 mg tissue/hr)	% change	GSH (nmole/100 mg tissue)	% change
Normal	20.00 ± 1.17^b^	-	73.90 ± 5.08^a^	-
Diabetic control	32.26 ± 2.75^a^	61.30	52.06 ± 0.73^b^	-29.55
Diabetic treated with Enalapril	19.32 ± 3.19^b^	-40.11	70.15 ± 7.40^a^	34.94
Diabetic treated with Paricalcitol	17.87 ± 1.04^b^	-44.60	48.94 ± 0.63^b^	-5.99
Diabetic treated with Enalapril and Paricalcitol	20.46 ± 1.06^b^	-36.57	71.58 ± 3.47^a^	37.49
F-probability	P<0.001		P<0.001	
LSD at 5% level	6.230		12.609	
LSD at 1% level	8.429		17.059	

- Data are expressed as mean ± SE. Number of detected samples in each group is six.

- Means, which share the same superscript symbol(s) are not significantly different.

- Percentage changes were calculated by comparing diabetic control group with normal control group and diabetic treated groups with diabetic control group.

**Table 4:** Effects of enalapril and paricalcitol on kidney GST and catalase activities in diabetic rats.

	GST (U/g tissue)	% change	Catalase (U/g tissue)	% change
Normal	1350.00 ± 9.31^a^	-	6.83 ± 0.17^a^	-
Diabetic control	838.50 ± 8.65^c^	-37.88	3.71 ± 0.28^d^	-45.68
Diabetic treated with Enalapril	953.55 ± 4.03^bc^	13.72	5.22 ± 0.61^b^	40.70
Diabetic treated with Paricalcitol	1204.50 ± 8.89^a^	43.64	4.14 ± 0.23^cd^	11.59
Diabetic treated with Enalapril and Paricalcitol	1138.46 ± 9.27^ab^	35.77	5.07 ± 0.25^bc^	36.65
F-probability	P<0.01		P<0.001	
LSD at 5% level	241.36		1.014	
LSD at 1% level	326.54		1.371	

- Data are expressed as mean ± SE. Number of detected samples in each group is six.

- Means, which share the same superscript symbol(s) are not significantly different.

- Percentage changes were calculated by comparing diabetic control group with normal control group and diabetic treated groups with diabetic control group.

On the other hand, the elevated fasting and postprandial serum glucose levels in diabetic rats were significantly improved (p<0.01; LSD) as a result of treatments with enalapril, paricalcitol and their combination. The diabetic group treated with enalapril and paricalcitol together exhibited the most potent effects in ameliorating the elevated serum fasting and postprandial glucose level; the recorded percentage changes were -63.55 and -60.37% respectively (Table 5). The serum insulin and C-peptide levels were significantly decreased in diabetic rats recording percentage changes of -76.98 and -83.64% respectively. The treatment with enalapril and/or paricalcitol successfully led to a significant increase of the lowered serum insulin and C-peptide levels. The treatment with enalapril was the most potent in increasing serum insulin and C-peptide levels recording percentage increases of 517.05 and 83.64% respectively (Table 6).

**Table 5:** Effects of enalapril and paricalcitol on serum fasting and postprandial glucose levels in diabetic rats.

	Fasting serum glucose (mg/dl)	% change	Postprandial serum glucose (mg/dl)	% change
Normal	70.01 ± 5.91^d^	-	98.00 ± 5.34^d^	-
Diabetic control	229.40 ± 12.56^a^	231.26	255.75 ± 7.67^a^	160.96
Diabetic treated with Enalapril	135.24 ± 6.91^b^	-41.04	190.66 ± 16.10^b^	-25.45
Diabetic treated with Paricalcitol	103.60 ± 5.09^c^	-54.83	157.41 ± 11.93^c^	-38.45
Diabetic treated with Enalapril and Paricalcitol	83.6 ± 3.66^cd^	-63.55	101.33 ± 2.56^d^	-60.37
F-probability	P<0.001		P<0.001	
LSD at 5% level	21.806		29.009	
LSD at 1% level	29.502		39.247	

- Data are expressed as mean ± SE. Number of detected samples in each group is six.

- Means, which share the same superscript symbol(s) are not significantly different.

- Percentage changes were calculated by comparing diabetic control group with normal control group and diabetic treated groups with diabetic control group.

**Table 6:** Effects of enalapril and paricalcitol on serum insulin and C-peptide levels in diabetic rats.

	Insulin (ng/ml)	% change	C-peptide (ng/ml)	% change
Normal	2.633 ± 0.111^a^	-	4.733 ± 0.264^a^	-
Diabetic control	0.606 ± 0.189^c^	-76.98	0.774 ± 0.109^c^	-83.64
Diabetic treated with Enalapril	1.833 ± 0.152^b^	202.47	4.776 ± 0.097^a^	517.05
Diabetic treated with Paricalcitol	1.413 ± 0.195^b^	133.16	4.200 ± 0.146^a^	442.63
Diabetic treated with Enalapril and Paricalcitol	1.640 ± 0.204^b^	170.62	3.240 ± 0.427^b^	317.70
F-probability	P<0.001		P<0.001	
LSD at 5% level	0.5063		0.7084	
LSD at 1% level	0.6850		0.9854	

- Data are expressed as mean ± SE. Number of detected samples in each group is six.

- Means, which share the same superscript symbol(s) are not significantly different.

- Percentage changes were calculated by comparing diabetic control group with normal control group and diabetic treated groups with diabetic control group.

**Fig 1:**
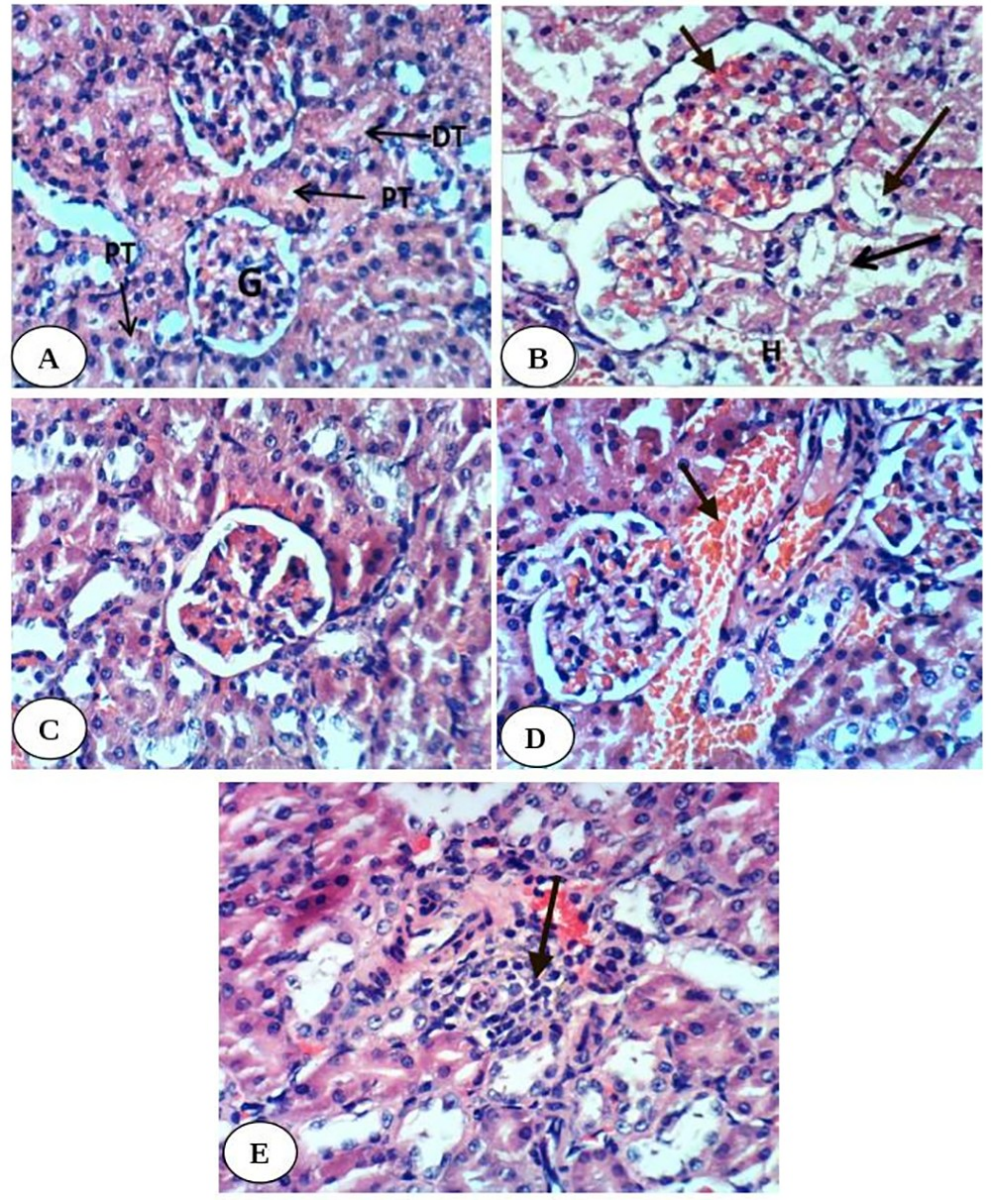
Photomicrographs of H & E stained kidney sections. Fig 1A is showing normal kidney histological structure with normal glomerulus (G), normal proximal tubule (PT) and normal distal tubule (DT). Fig 1B is showing severe diffuse tubular necrosis (long arrow), severe congestion of glomerular tuft (short arrow) and intertubular hemorrhage (H) in diabetic group. Fig 1C is showing normal histological structure in diabetic group treated with enalapril. Fig 1D is showing congestion of blood vessel (arrow) in diabetic group treated with paricalcitol. Fig 1E is showing focal necrosis and inflammatory cell infiltration (arrow) in diabetic rats treated with both enalapril and paricalcitol. (H&E; 400X).

Histopathological examination of kidney tissues of diabetic group showed severe necrosis of epithelial cells lining renal tubules, intense congestion of glomerular tuft (Fig 1B) and intertubular hemorrhage as compared with kidney histological structure of normal group (Fig 1A). The kidney section of diabetic group treated with enalapril showed no histopathological changes (Fig 1C) while mild congestion of glomerular tuft and intense congestion of blood vessels were observed in diabetic rats treated with paricalcitol. Moreover, although there are inflammatory cells in diabetic group treated with a combination of both treatments, most of tubules appeared with normal intact architecture (Fig 1D).

**Fig 2:**
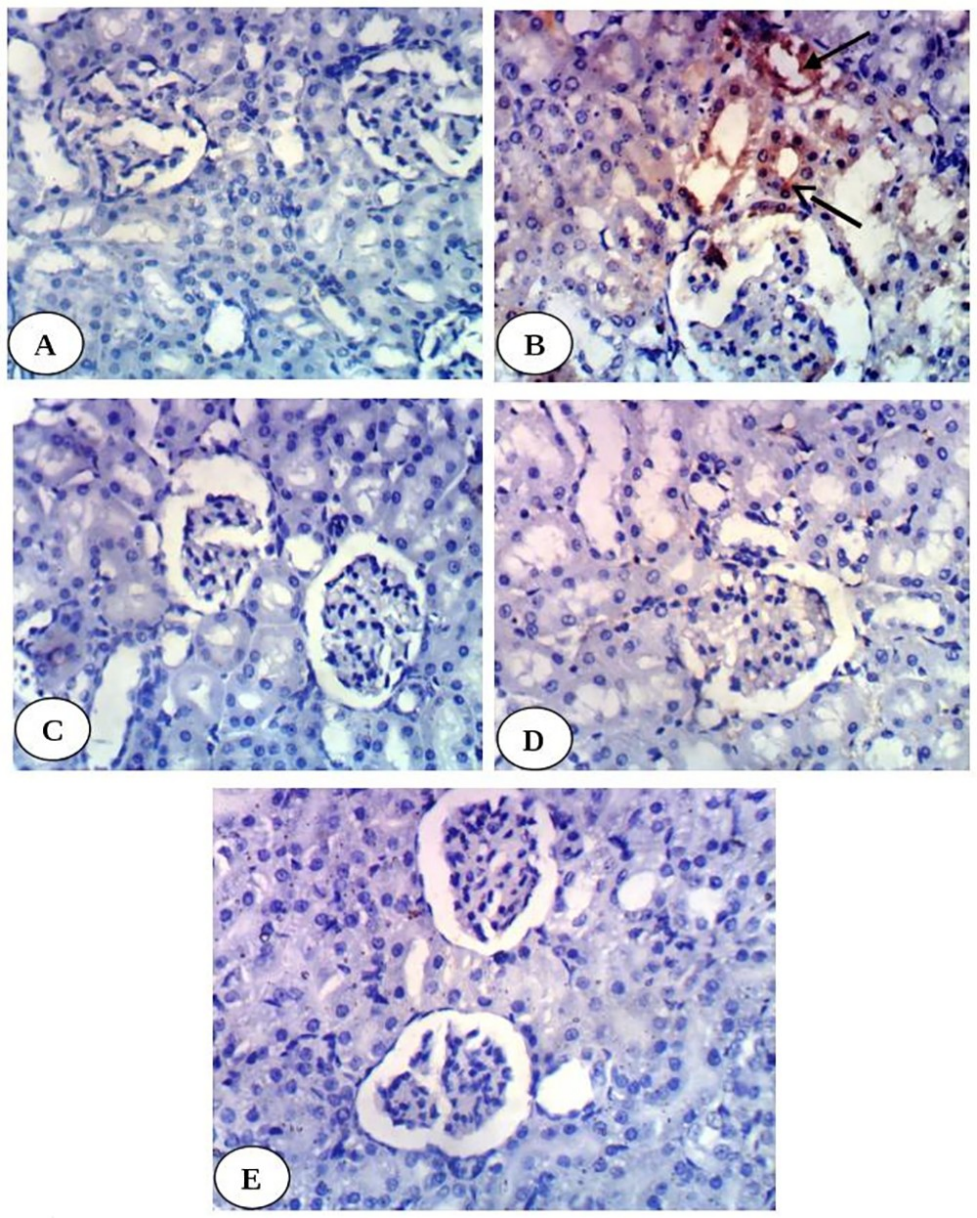
Photomicrographs of immunohistochemical staining of TNF-α in kidney tissues showing a very weak expression in normal group (A), diabetic groups treated with enalapril (C), paricalcitol (D) and their combination (E) while strong positive expression in diabetic control (B). (400X).

**Fig 3:**
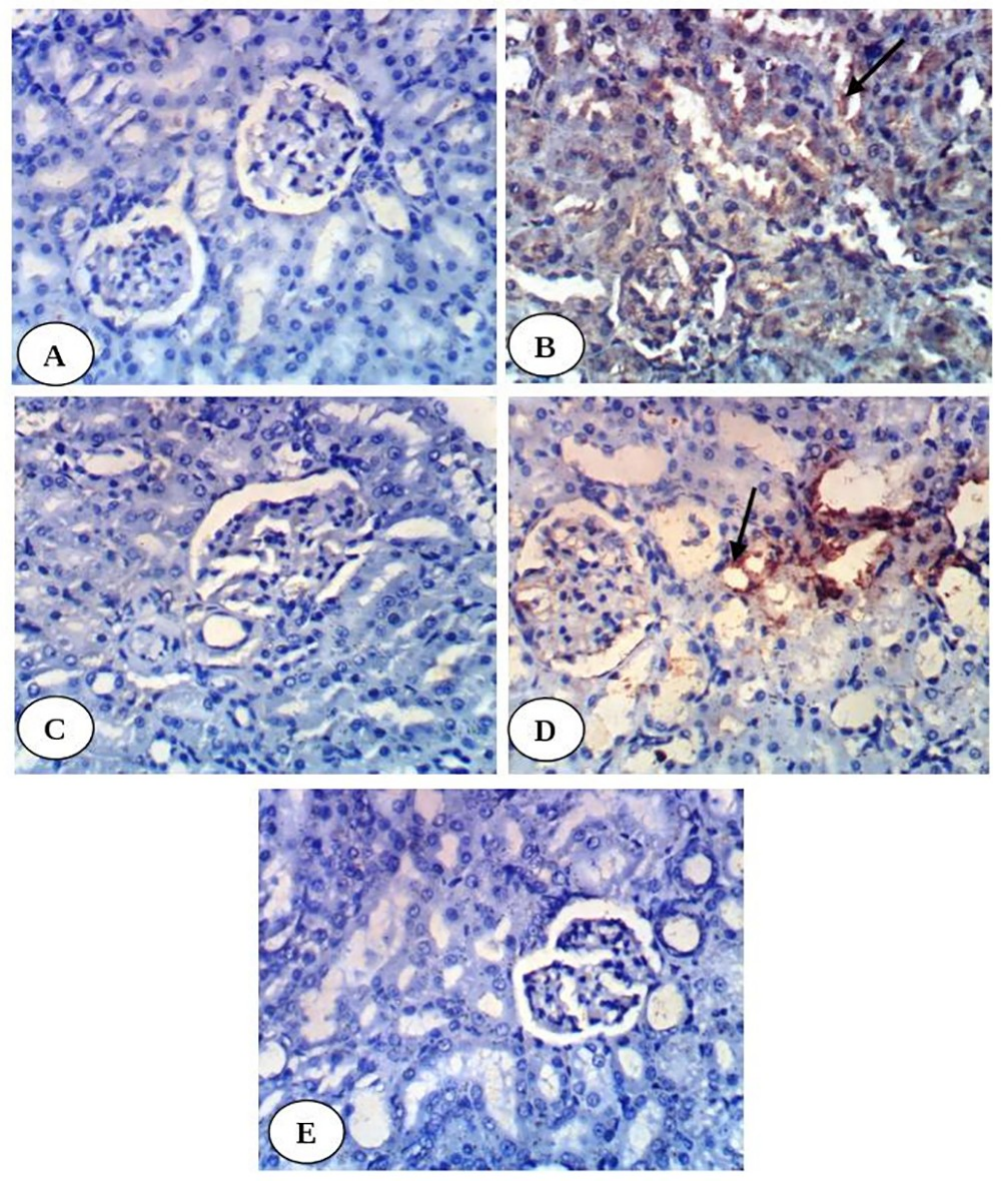
Photomicrographs of immunohistochemical staining of p53 in kidney tissues showing a very weak expression in normal group (A), diabetic groups treated with enalapril (C) and its combination with paricalcitol (E) while moderate expression in diabetic rats treated with paricalcitol (D) and strong positive expression in diabetic control group (B). (400X).

**Fig 4:**
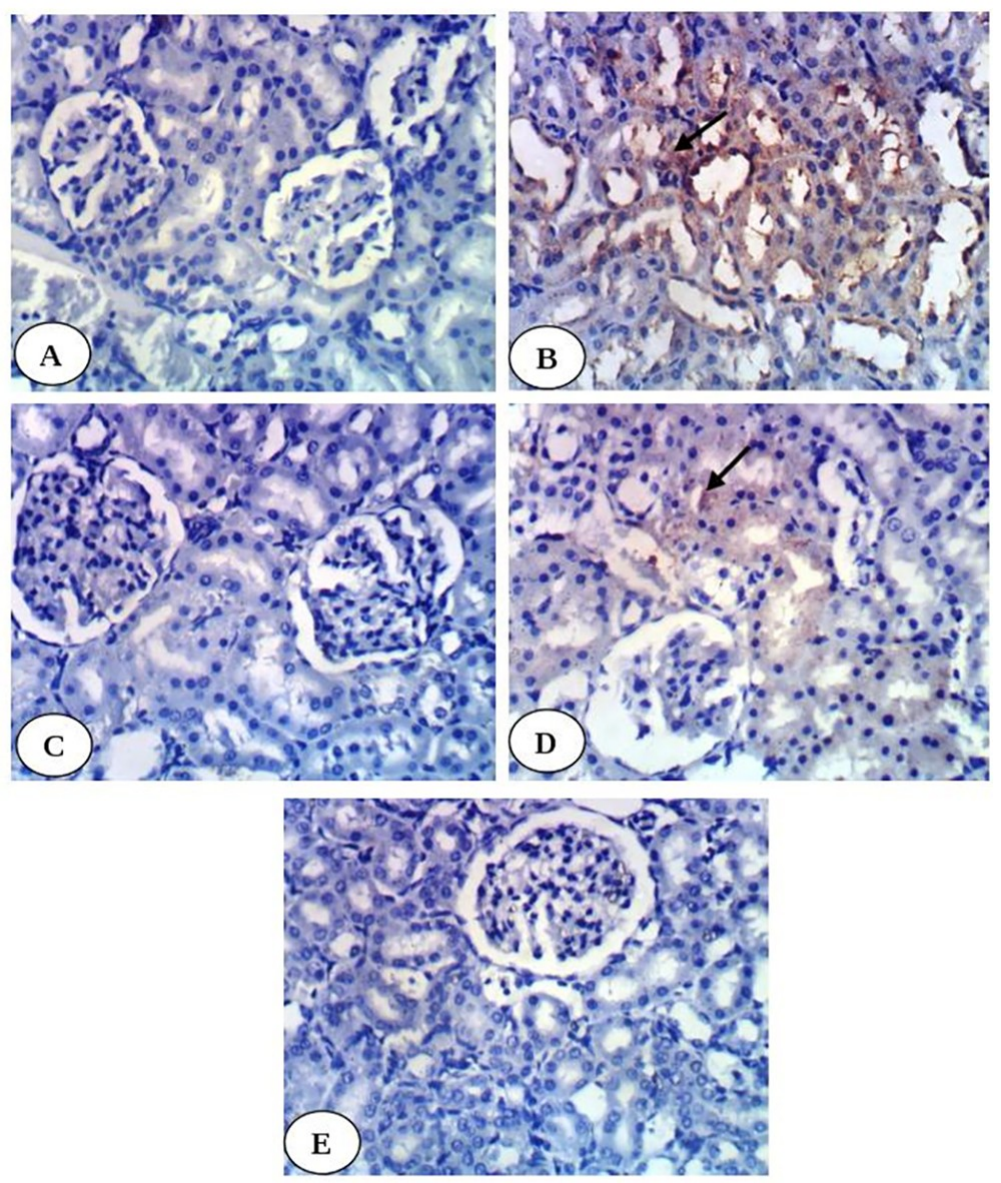
Photomicrographs of immunohistochemical staining of caspase-3 in kidney tissues showing a very weak expression in normal group (A), diabetic group treated with enalapril (C) and its combination with paricalcitol (E) while strong positive expression in diabetic control (B) and mild expression in diabetic group treated with paricalcitol (D). (400X).

**Fig 5:**
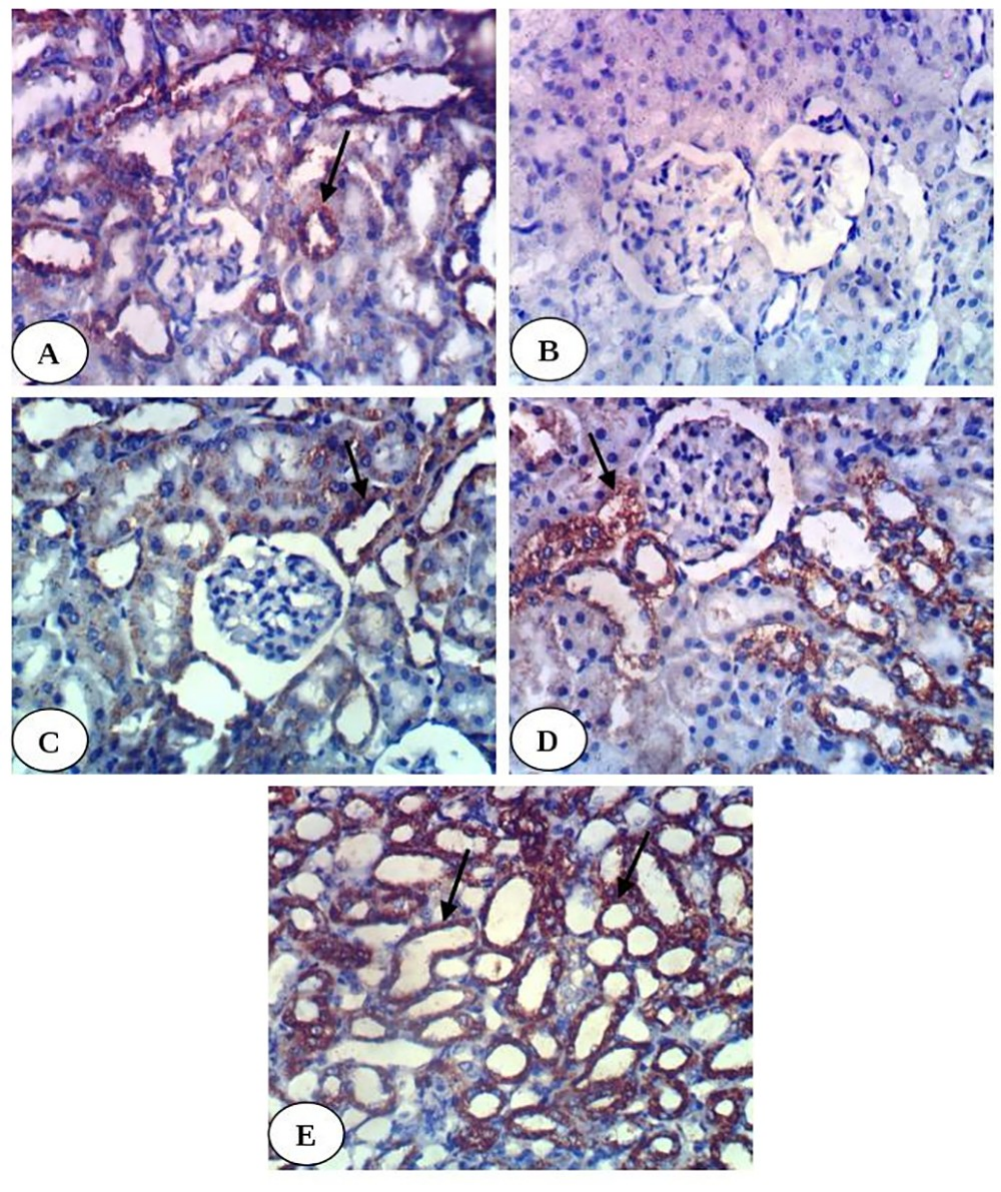
Photomicrographs of immunohistochemical staining of Bcl-2 in kidney tissues showing a weak expression in diabetic control group (B) and strong positive expression in normal group (A), diabetic groups treated with enalapril (C), paricalcitol (D) and their combination (E). (400X).

Immunohistochemical study revealed weak expression of kidney TNF-α in normal control group (Fig 2A) and in diabetic groups treated with enalapril (Fig 2C), paricalcitol (Fig 2D) and their combination (Fig 2E) while it showed intense expression in diabetic control group (Fig 2B). Kidney p53 exhibited very weak expression in normal group (Fig 3A), diabetic groups treated with enalapril (Fig 3C) and its combination with paricalcitol (Fig 3E) while it showed moderate expression in diabetic rats treated with paricalcitol (Fig 3D) and strong positive expression in diabetic control group (Fig 3B). Kidney caspase-3 exhibited very weak expression in normal group (Fig 4A), diabetic group treated with enalapril (Fig 4C) and its combination with paricalcitol (Fig 4E) while it showed strong positive expression in diabetic control (Fig 4B) and mild expression in diabetic group treated with paricalcitol (Fig 4D). Conversely, kidney Bcl-2 showed weak expression in diabetic control group (Fig 5B) and strong positive expression in normal group (Fig 5A), diabetic groups treated with enalapril (Fig 5C), paricalcitol (Fig 5D) and their combination (Fig 5E). The results of imageJ analysis indicated that the integrated staining intensity (Table 7) and the percent stained area (Table 8) of kidney TNF-α, p53 and caspase-3 showed a significant increase in diabetic rats while those of kidney Bcl-2 depicted a significant decrease as compared with normal rats. As a result of treatment of diabetic rats with enalapril, paricalcitol and their combination, the integrated staining intensity and the percent stained area exhibited a significant decrease for kidney TNF-α, p53 and caspase-3 and a significant increase for Bcl-2. The effect of treatment with enalapril and its combination with paricalcitol was more significantly (p<0.05) potent in decreasing p53 and caspase-3 contents as compared with the diabetic group treated with paricalcitol alone. Moreover, the diabetic rats treated with enalapril and paricalcitol in combination exhibited no further significant effect (p>0.050) on expressed renal p53 and caspase-3 as compared with the diabetic group treated with enalapril alone. On the other hand, the expressed Bcl-2 in kidney of the diabetic group treated with enalapril and paricalcitol in combination showed a significant decrease (p<0.05) when compared with the diabetic groups singly treated with either enalapril or paricalcitol (Tables 7 and 8).

**Table 7:** Immunohistochemical staining integrated intensity (.10^6^) for the expression of TNF-α, p53, caspase-3 and Bcl-2 in kidney of normal, diabetic control and diabetic groups treated with enalapril, paricalcitol and their mixture.

	TNF-α	p53	Caspase-3	Bcl-2
Normal	0.194±0.043^b^	1.076±0.157^c^	0.349±0.046^c^	17.320±1.024^b^
Diabetic control	5.848±0.659^a^	29.628±4.688^a^	10.190±2.241^a^	2.303±0.214^c^
Diabetic treated with Enalapril	0.370±0.143^b^	1.107±0.335^c^	0.660±0.209^c^	15.396±1.499^b^
Diabetic treated with Paricalcitol	1.040±0.342^b^	10.151±1.581^b^	5.704±0.733^b^	12.421±4.078^bc^
Diabetic treated with Enalapril and Paricalcitol	0.268±0.083^b^	1.406±0.342^c^	0.389±0.083^c^	30.224±7.056^a^
F-probability	P<0.001	P<0.001	P<0.001	P<0.01
LSD at 5% level	1.074	7.009	3.338	11.768
LSD at 1% level	1.528	9.969	4.748	16.739

- Data are expressed as mean ± SE. Number of replicates in each group is 3.

- The integrated intensities were calculated for positive immunohistochemical reactions in a standard measuring frame of the captured images,

- Means, which share the same superscript symbol(s) are not significantly different.

**Table 8:** Stained area percent for the expression of TNF-α, p53, caspase-3 and Bcl-2 in kidney of normal, diabetic control and diabetic groups treated with enalapril, paricalcitol and their mixture.

	TNF-α	p53	Caspase-3	Bcl-2
Normal	0.096±0.022^b^	0.654±0.140^c^	0.342±0.169^c^	8.637±0.511^b^
Diabetic control	2.916±0.328^a^	14.774±2.338^a^	5.082±1.118^a^	1.148±0.107^c^
Diabetic treated with Enalapril	0.195±0.070^b^	0.553±0.167^c^	0. 330±0.010^c^	7.678±0.747^b^
Diabetic treated with Paricalcitol	0.518±0.170^b^	5.062±0.788^b^	2.844±0.365^b^	8.448±1.335^b^
Diabetic treated with Enalapril and Paricalcitol	0.133±0.413^b^	0.701±0.171^c^	0.194±0.042^c^	15.121±3.518^a^
F-probability	P<0.001	P<0.001	P<0.001	P<0.01
LSD at 5% level	0.535	3.498	1.675	5.455
LSD at 1% level	0.761	4.976	2.382	7.759

- Data are expressed as mean ± SE. Number of replicates in each group is 3.

- Means, which share the same superscript symbol(s) are not significantly different.

**Fig 6:**
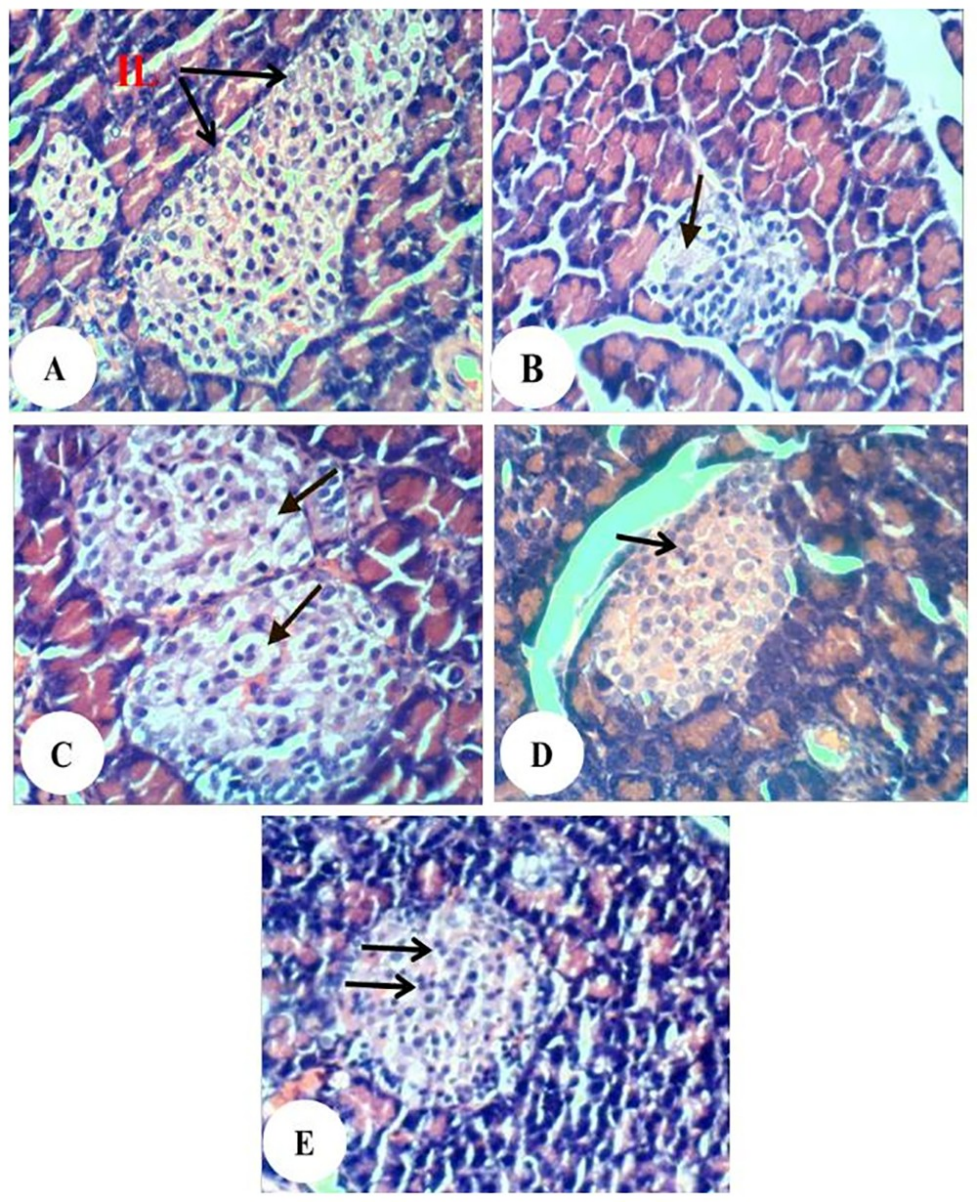
Photomicrographs of pancreas sections showing no histopathological changes in normal group (A), necrosis (arrow) and decrease in the size of islets of diabetic control group (B), marked increase of islet size and vacuolations (arrow) in diabetic group treated with enalapril (C), intact islet in diabetic group treated with paricalcitol (D) and relatively large-sized islets with highly active divided cells in diabetic group treated with a combination of enalapril and paricalcitol (E). (H&E; 400X).

The pancreas sections of normal rats showed normal islet histological architecture and integrity (Fig 6A). The islets of Langerhans of diabetic rats exhibited necrotic and destructive changes of islets cells and decrease in the size of islets (Fig 6B) as compared with normal islets. The diabetic rats treated with enalapril showed marked increase of islet size but still vacuolations are present (Fig 6C). The diabetic rats treated with paricalcitol showed intact islet with normal architecture (Fig 6D). The diabetic rats treated with a combination of enalapril and paricalcitol exhibited relatively large-sized islets with highly active divided cells (Fig 6E).

## Discussion

DN, a severe complication of diabetes mellitus, is the most common cause of end stage renal failure. About 15–25% of type 1 diabetes patients and 30–40% of patients with type 2 diabetes suffer from DN [4]. In this context, the model of type 1 diabetes, rat STZ-induced diabetes model, is used in the present study to investigate the pathogenesis of DN [32]. STZ is an agent of choice to induce experimental diabetes mellitus due to its ability to induce specific necrosis of the pancreatic beta cells that results in degranulation and loss of capacity to secrete insulin [33], thereby leading to hyperglycemia and diabetic complications such as nephropathy [32]. As indicated in the present study, STZ induces a significant increase in fasting and post-prandial serum glucose levels, a significant depletion in serum insulin and C-peptide levels, decrease in the size of islets and necrotic changes in the islet cells and subsequent kidney dysfunction.

**Fig 7:**
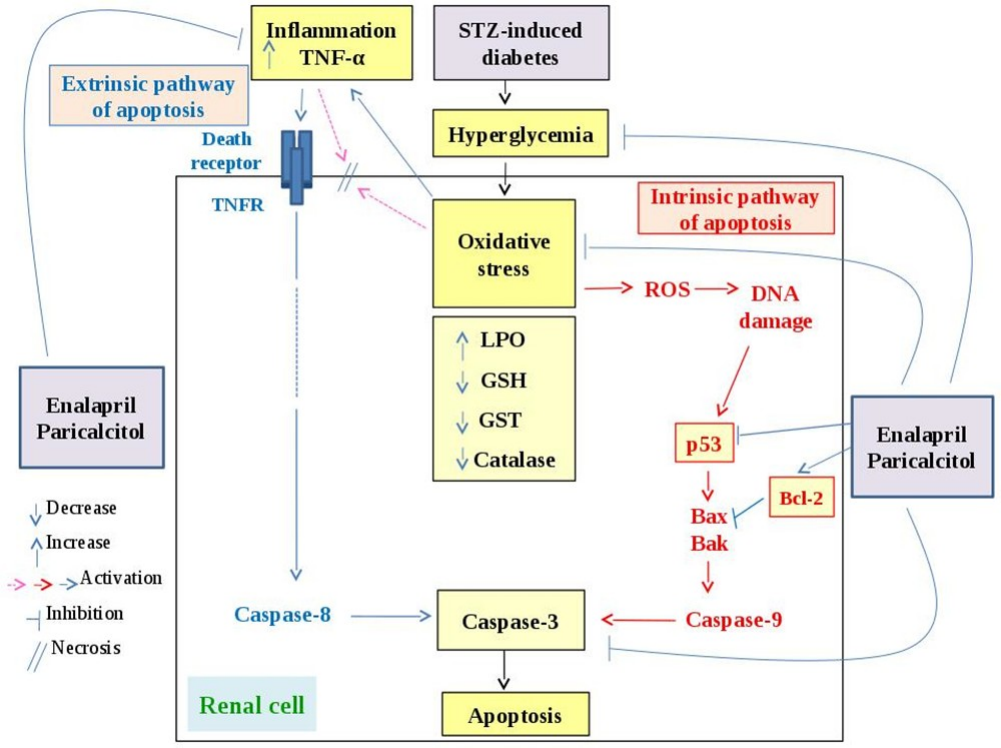
Schematic diagram of the effects of STZ-induced diabetes and hyperglycemia on oxidative stress, inflammation and apoptosis and the mechanisms of actions of enalapril and paricalcitol to suppress these processes.

In the present study, the STZ-induced diabetic rats exhibited impairment in kidney function that was manifested by a significant elevation of serum urea, uric acid, creatinine, sodium and potassium levels as well as derangement in kidney histological architecture and integrity which was marked by severe glomerular congestion, tubular necrosis and intertubular hemorrhage. These results are in accordance with Ahmed [34] who reported a significant increase in serum urea, uric acid and creatinine levels tandem to severe hyperemia in the glomerular tufts, intertubular hyperemia and degenerative changes in the epithelium cells lining the renal tubules in STZ-induced diabetic rats. The damaging effects of STZ on kidney may be attributed to the increased oxidative stress and the attenuated antioxidant defense system. The present study supports this attribution since the STZ-induced diabetic rats exhibited a significant elevation in the kidney LPO and a significant decrease in kidney GSH content as well as suppression of GST and catalase activities. In agreement with these results, de Brito Amaral *et al*. [35] revealed a significant increase in kidney LPO in sedentary and trained STZ-induced diabetic rats as compared with normal control. Moreover, Ziamajidi *et al*. [36] confirmed the elevation of LPO and total oxidative stress in nicotinamide (NA)/STZ-induced diabetic rats. In our opinion, it can be stated that the persistent hyperglycemia in diabetes mellitus leads to increased production of reactive oxygen species (ROS) which are involved in the etiology of several diabetic complications including DN. ROS deplete the antioxidant defenses of the cell thus making it more susceptible to oxidative damage [37]. ROS further target lipid, DNA and protein leading to their oxidation which further leads to changes in cellular structure and function [38] such as renal cell necrosis and mutation in genes that control regulatory proteins of cell proliferation and apoptosis (Fig 7).

In addition to the role of oxidative stress in inducing the renopathy, the inflammation and apoptosis may have an important role in eliciting kidney dysfunction and histological deteriorations since the STZ-induced diabetic rats, in the present study, exhibited a remarkable increase in the expression of immunohistochemically-detected pro-inflammatory cytokine, TNF-α and apoptotic markers including p53 and caspase-3 as well as a decrease in the anti-apoptotic markers Bcl-2. These results are in concurrence with the previous study of Pradeep and Srinivasan [39] who demonstrated an increased apoptotic mediator, Bax and decrease in Bcl-2, detected by immunoflourscence and immunohischemical techniques, in kidney of STZ-induced diabetic rats. The present study is also in accordance with Sha *et al*. [40] who elucidated that apoptosis may contribute to the development of STZ-induced DN in rats.

The treatment of diabetic rats with enalapril and/or paricalcitol, in the present study, resulted in a marked improvement of kidney function represented by a significant decrease in the elevated serum urea, uric acid and creatinine levels along with a remarkable amelioration of the deteriorated kidney histological changes. These ameliorations in kidney function and histological architecture and integrity are associated with the improvements in the glycemic state, serum insulin and C-peptide levels, islets histological changes, kidney oxidative stress and antioxidant defense system, kidney TNF-α as pro-inflammatory cytokine as well as kidney apoptotic mediators (p53 and caspase-3) and anti-apoptotic marker (Bcl-2). It is related here to mention that although the treatment with enalapril and paricalcitol in combination produced the most potent effect on the elevated serum glucose levels and the lowered kidney Bcl-2 in diabetic rats, it caused no further additional significant effects on serum insulin and C-peptide, serum parameters of kidney function, kidney antioxidant defense system as well as renal TNF-α, p53 and caspase-3 when compared to the diabetic group treated with enalapril alone.

The more enhanced improvement effects on fasting and post-prandial glucose levels by the treatment of diabetic rats with enalapril and paricalcitol in combination in spite of non-significant further increase in serum insulin and C-peptide levels as compared with the diabetic rats treated singly with enalapril or paricalcitol led us to suggest that the combined treatment may be more effective in improving tissue insulin sensitivity. These results are in accordance with Suarez-Martinez *et al*. [41] who reported that the plasma level of adiponectin, a key effector in insulin sensitivity, increased 34%, 73% and 81% in ApoE-deficient mice treated with paricalcitol, enalapril and combination of the two drugs respectively.

In concurrence with the present study, Agrawal *et al*. [42] revealed that enalapril has potent anti-hyperglycemic effect in alloxan-induced diabetic rats. Recent clinical trials indicated that agents which interrupt the renin-angiotensin axis such as enalapril reduce the risk of developing diabetes compared with other classes of antihypertensive drugs and blockage of the effects of angiotensin II might improve the blood pressure and the rate of blood flow to insulin-sensitive tissues [43]. Moreover, interruption of RAAS could lead to improved insulin signaling and thereby tissue insulin sensitivity [43]. Many other publications have reported that RAAS induced islet fibrosis, inflammation, oxidative stress, and impaired insulin secretion whereas RAAS blockade could improve islet morphology and function and increased glucose tolerance [44,45]. Thus, based on these publications and the result of the present study, we can suggest that the antihyperglycemic effects of enalapril in STZ-induced diabetic rats may be attributed to the enhanced insulin secretion from β-cells of islets of Langerhans and improved tissue insulin sensitivity as well.

In addition to its improving effect on the pancreatic islets and increasing effects on the β-cells number as indicated in the present study, paricalcitol (as well as vitamin D analogs) may act through VDR-mediated modulation of expression of calbindin-D (28k) that activates intracellular calcium flux in the islet cells leading to increase in insulin secretion and release [46–48]. Furthermore, the enhanced expression of calbindin-D(28k) in pancreatic islet beta-cell protects against cytokine (IL-1β, TNF-α and interferon gamma [INF-γ])-induced apoptosis and necrosis probably by inhibiting the stimulatory effects of the cytokines on oxidative stress and free radical formation [49]. In our opinion, the improvement in the glycemic state secondary to the increase in insulin secretion (or enhanced insulin sensitivity) as a result of treatment of diabetic rats with enalapril and/or paricalcitol may result in a decrease in the oxidative stress that may in turn leads to improvements in kidney function and structural integrity (Fig 7).

The pathogenesis of renal injury included complex pathway crosstalk contributing to the increased oxidative stress and inflammation, as well as renal tubular apoptosis, during the disease course [50]. As STZ diabetes-induced oxidative stress, local inflammation and tubular apoptosis were implicated in the pathogenesis of renal dysfunction as confirmed in this study and previous publications [35,36,39,40], the improvement effects of enalapril and/or paricalcitol on these processes in treated STZ-induced diabetic rats may be all involved to alleviate the kidney function and kidney structural integrity. In this way, enalapril, a non-sulfhydryl ACEI, has shown renoprotective effect in various animal models including DN animal model as reported by previous publication [51]. Clinically, enalapril exhibited renoprotective effect in patients with CKD, DN and hypertension after renal transplantation [52,53]. In parallel to the present study, Hou *et al*. [54] stated that the enalapril maleate attenuated renal injury in a multiple drugs-induced nephropathy rat model by exerting renal vasodilatory, antioxidation, antiapoptotic, and endothelial function-promoting effects. On the other hand, Yang *et al*. reported apoptosis inhibition in case of STZ diabetic rats after observing higher levels of insulin and C-peptide in diabetic group receiving active vitamin D3 (1,25(OH)2D3) [55]. Vitamin D may also mitigate kidney damage by overwhelming fibrosis, inflammation, and apoptosis, through hindering multiple pathways including RAAS, the nuclear factor-κB (NF-κB), the transforming growth factor-β (TGF-β)/Smad, and the Wnt/β-catenin signaling pathways [56,57,58]. It is also worth mentioning here that VDR activated by vitamin D and vitamin D analogs are found originally in the classic vitamin D target organs involved in mineral homeostasis such as the intestine, bone, kidney, and parathyroid gland as well as in many other tissues and cells types including β-cells, smooth muscle cells and cardiomyocytes [46]. Paricalcitol, a better identified VDR activator, was stated to inhibit renal inflammatory cells infiltration by promoting VDR mediated sequestration of NF-κB signaling and to decrease pro-inflammatory cytokines and oxidative stress [47].

The increased LPO in the renal tissue observed in diabetic rats, in present study, was significantly suppressed as a result of treatment with enalapril and/or paricalcitol while the lowered GSH content as well as catalase and GST activities were increased, thus suggesting the antioxidant capacity of paricalcitol and enalapril. Thus, the decreased LPO after treatment with enalapril and/or paricalcitol, in the present study, could be due to the enhancement of the antioxidant defense system. In parallel with the present results, it was reported by the previous publications that enalapril treatment, on account of its antioxidant properties, reduced oxidative stress in the kidney tissues of spontaneously hypertensive rats [59] and STZ-induced diabetic rats [51]. The present results are also in consistence with a previous study of the effect of paricalcitol in hemodialysis patients that had revealed a similar effect of paricalcitol on catalase enzyme [17].

Consequentially, glomerular and tubular TNF-α, in the present study, showed increased expression in diabetic kidney and exhibited a decreased expression in diabetic rats treated with enalapril and/or paricalcitol reflecting the anti-inflammatory effect of these drugs; the effect of enalapril and paricalcitol in combination was not significantly potent when compared with groups treated with enalapril or paricalcitol. These results are in concordance with Navarro *et al*. [60] who stated that enalapril administration nearly completely abolished the increase in renal TNF-α messenger RNA expression and reduced urinary albumin excretion in STZ-induced diabetic rats reflecting the role of anti-inflammatory effect in the improvement of kidney function. Those authors also found that blockade of RAAS by enalapril prevent the enhanced expression of TNF-α suggesting the possible regulatory role of RAAS on renal inflammatory status. The present results also go parallel with Izquierdo *et al*. [17] who found that after paricalcitol treatment of patients with renal disease, levels of the inflammatory markers CRP, TNF-α, IL-6 and IL-18 were significantly reduced in serum and the level of anti-inflammatory cytokine IL-10 was increased. The anti-inflammatory possessions of active vitamin D and its analogues (such as paricalcitol) as well as enalapril may be endorsed to their ability to overwhelm the NF-κB pathway, a key transcription factor that is supposed to facilitate acute and chronic inflammation by regulating gene expression of cytokines and chemokines (including interleukin-6 and tumor necrosis factor-α) [61]. NF-κB activation associated with increased ROS generation is pivotal in the consequent expression of pro-inflammatory cytokines like TNF-α. These chemokines may then facilitate migration and infiltration of inflammatory cell and a secondary wave of ROS generation, and further amplify the inflammatory cascade and injury [62]. In another way, paricalcitol weakens renin and angiotensin II expression in VDR knockout mice [63], telling that paricalcitol inhibits renal inflammation by overwhelming the RAAS, as angiotensin II is a known pro-inflammatory stimulus. Taken together, these findings indicate that enalapril and vitamin D analogue, paricalcitol, might be useful for treating inflammatory kidney diseases.

In trial to elucidate the role of apoptosis in the pathogenesis of DN and in the ameliorative effects of enalapril and paricalcitol, renal apoptotic proteins including p53 and caspase-3, and anti-apoptotic protein, Bcl-2, were immunohistochemically detected. The present study indicated that the expression of tubular p53 and caspase-3 were remarkably increased in diabetic rats and were decreased as a result of treatment with enalapril and/or paricalcitol. The effect of enalapril alone and enalapril concomitant with paricalcitol seemed to be more potent in decreasing the expression of tubular p53 and caspase-3 than the effect of paricalcitol alone. Moreover, the combined effect of two drugs was not significant as compared with the group treated with enalapril alone. The tubular expression of anti-apoptotic protein Bcl-2 exhibited a reverse pattern of changes with p53 and caspase-3. The treatment with enalapril and paricalcitol in combination was the most effective in increasing Bcl-2. All these observations indicate a protective effect of paricalcitol and enalapril on STZ-induced renal tubular apoptosis probably *via* modifying expression of p53, Bcl-2 family proteins and caspase-3 (Fig 7).

The p53 protein corresponds to a number of processes associated with the life and death of the cell. It regulates the repair of cellular DNA and encourages apoptosis when the damage of the gene is too serious and it is difficult to repair [64]. It has also been established that the p53 protein, as a consequence of stress factors, crosses into the mitochondria and triggers the expression of pro-apoptotic genes, for example Puma, Bax, Apaf-1, Noxa. In addition, it inhibits the expression of anti-apoptotic genes, like those of the Bcl-2 family (Bcl-2, Bcl-X, Bcl-in, Mcl-1); this evidence was confirmed in the present investigation since the tubular expression of anti-apoptotic protein Bcl-2 exhibited a reverse pattern of changes with p53. The p53 protein together with these pro-apoptotic proteins, are moved into the mitochondria where they encourage an upsurge in the permeability of mitochondrial membranes and the release of cytochrome C, which attaches to the apoptotic protease activating factor 1 (Apaf-1) and with the caspase-9 proenzyme, to generate the complex called the "apoptosome". The apoptosome, consecutively, produces activation of caspase-9. The later consequently stimulates the caspase-3 proenzyme activation to the protease stage, which then sticks to the effector caspase group. These caspases induce intracellular protein lysis and morphological distinctive changes of apoptosis [64]. In this regard, it was reported that apoptosis could aggravate the pathogenesis of nephrotoxicity *via* expression of caspase-3 [65] which is activated in the apoptotic cell both by extrinsic (death ligand such as TNF-α) and intrinsic (high production of mitochondrial ROS) pathways [66,67] as indicated in our proposed Fig 7. It is relevant to mention that the TNF-α-induced cell death is predominantly apoptotic but may also occur by necrosis [68] as indicated in our designed Fig 7. Mitochondrial oncogene product, Bcl-2, prevented caspase-3 activation during a variety of proapoptotic conditions [69].

The antiapoptotic effect of enalapril observed in the current study was also in accordance with the study of Rani *et al*. [70] who revealed that pretreatment with enalapril dose dependently restored the cisplatin-induced apoptosis towards normal as it reduced Bax, caspase-3, cytochrome C, and p53 expressions with concomitant increase in the Bcl-2 expression, thus resulting in anti-apoptotic potential. The antiapoptotic effects of paricalcitol were also reported. A previous study showed that paricalcitol reduces the increased expression of phospho-p53 which recruits apoptotic processes in a cisplatin-induced rat model [71]. Moreover, it was reported that an increased Bax/Bcl-2 ratio and the cleaved form of caspase-3, which are apoptotic markers, are reversed by paricalcitol treatment in gentamicin-induced kidney injury [72]. Thus, the enalapril and paricalcitol have anti-apoptotic effects and p53, caspase-3 and Bcl-2 may have crucial role to mediate these effects.

## Conclusions

The study concluded that paricalcitol and/or enalapril potentially protect against STZ-induced diabetic renopathy *via* their potencies to improve the diabetic condition, suppress the oxidative stress, enhance the antioxidant defense system and decrease the apoptosis through attenuating the renal expression of p53 and caspase-3 and enhancing the expression of Bcl-2. Moreover, although the treatment of diabetic rats with enalapril and paricalcitol in combination produced the most potent effects on serum glucose levels and renal Bcl-2 expression, it did not add any further significant improvement effects on kidney function and kidney histological integrity as compared with the diabetic groups singly treated with either enalapril or paricalcitol.

## Supporting information

S1 Table Effects of enalapril and paricalcitol on serum urea, uric acid and creatinine levels in diabetic rats. (PDF)

S2 Table Effects of enalapril and paricalcitol on serum sodium and potassium levels in diabetic rats. (PDF)

S3 Table Effects of enalapril and paricalcitol on kidney GSH content and LPO in diabetic rats. (PDF)

S4 Table Effects of enalapril and paricalcitol on kidney GST and catalase activities in diabetic rats. (PDF)

S5 Table Effects of enalapril and paricalcitol on serum fasting and postprandial glucose levels in diabetic rats. (PDF)

S6 Table Effects of enalapril and paricalcitol on serum insulin and C-peptide levels in diabetic rats. (PDF)

S7 Table Immunohistochemical staining integrated intensity (.10^6^) for the expression of TNF-α, p53, caspase-3 and Bcl-2 in kidney of normal, diabetic control and diabetic groups treated with enalapril, paricalcitol and their mixture. (PDF)

S8 Table Stained area percent for the expression of TNF-α, p53, caspase-3 and Bcl-2 in kidney of normal, diabetic control and diabetic groups treated with enalapril, paricalcitol and their mixture. (PDF)
