# Supplementary material for: Effects of enalapril and paricalcitol treatment on diabetic nephropathy and renal expressions of TNF-α, p53, caspase-3 and Bcl-2 in STZ-induced diabetic rats
Source: PLoS One. 2019 Sep 17;14(9):e0214349. doi: 10.1371/journal.pone.0214349 (PMC6748411; doi:10.1371/journal.pone.0214349)
Supplement: S1 Table — (PDF) [file pone.0214349.s001.pdf]

Table 1: Effects of enalapril and paricalcitol on serum urea, uric acid and creatinine levels in diabetic rats

	Urea (mg/dl)	% change	Uric acid (mg/dl)	% change	Creatinine (mg/dl)	% change
Normal	29.17 ± 2.29[d]	-	1.31 ± 0.132[b]	-	0.64 ± 0.004[b]	-
Diabetic control	83.52 ± 9.79[a]	186.32	2.19 ± 0.295[a]	67.17	1.06 ± 0.138[a]	63.07
Diabetic treated with Enalapril	42.29 ± 1.54[cd]	-49.36	1.32 ± 0.181[b]	-39.72	0.62 ± 0.003[b]	-41.50
Diabetic treated with Paricalcitol	65.56 ± 5.15[b]	-21.50	1.52 ± 0.140[b]	-30.59	0.78 ± 0.082[b]	-26.41
Diabetic treated with Enalapril and Paricalcitol	55.67 ± 5.17[bc]	-33.34	1.74 ± 0.230[ab]	-20.54	0.70 ± 0.074[b]	-3369
F-probability	P<0.001		P<0.05		P<0.01	
LSD at 5% level	16.322		0.598		0.240	
LSD at 1% level	22.082		0.809		0.325	

- Data are expressed as mean ± SE. Number of detected samples in each group is six.
- Means, which share the same superscript symbol(s) are not significantly different.
- Percentage changes were calculated by comparing diabetic control group with normal control group and diabetic treated groups with diabetic control group.
