# Supplementary material for: Effects of enalapril and paricalcitol treatment on diabetic nephropathy and renal expressions of TNF-α, p53, caspase-3 and Bcl-2 in STZ-induced diabetic rats
Source: PLoS One. 2019 Sep 17;14(9):e0214349. doi: 10.1371/journal.pone.0214349 (PMC6748411; doi:10.1371/journal.pone.0214349)
Supplement: S2 Table — (PDF) [file pone.0214349.s002.pdf]

Table 2: Effects of enalapril and paricalcitol on serum sodium and potassium levels in diabetic rats

	**Sodium (mg/dl)**	**% change**	**Potassium (mg/dl)**	**% change**
Normal	149.19 ± 0.38[c]	-	4.90 ± 0.21[b]	-
Diabetic control	154.55 ± 0.84[a]	3.59	5.98 ± 0.26[a]	22.04
Diabetic treated with Enalapril	150.27 ± 0.12[bc]	-2.76	5.16 ± 0.04[b]	-13.71
Diabetic treated with Paricalcitol	151.84 ± 0.35[b]	-1.75	5.23 ± 0.35[b]	-12.54
Diabetic treated with Enalapril and Paricalcitol	151.59 ± 0.74[b]	-1.91	4.83 ± 0.15[b]	-19.23
F-probability	P<0.001		P<0.05	
LSD at 5% level	1.627		0.668	
LSD at 1% level	2.202		0.904	

- Data are expressed as mean ± SE. Number of detected samples in each group is six.
- Means, which share the same superscript symbol(s) are not significantly different.
- Percentage changes were calculated by comparing diabetic control group with normal control group and diabetic treated groups with diabetic control group.
