# Supplementary material for: Effects of enalapril and paricalcitol treatment on diabetic nephropathy and renal expressions of TNF-α, p53, caspase-3 and Bcl-2 in STZ-induced diabetic rats
Source: PLoS One. 2019 Sep 17;14(9):e0214349. doi: 10.1371/journal.pone.0214349 (PMC6748411; doi:10.1371/journal.pone.0214349)
Supplement: S3 Table — (PDF) [file pone.0214349.s003.pdf]

Table 3: Effects of enalapril and paricalcitol on kidney GSH content and LPO in diabetic rats.

	**LPO (nmole MDA/100 mg tissue/hr)**	**% change**	**GSH (nmole/100 mg tissue)**	**% change**
Normal	20.00 ± 1.17[b]	-	73.90 ± 5.08[a]	-
Diabetic control	32.26 ± 2.75[a]	61.30	52.06 ± 0.73[b]	-29.55
Diabetic treated with Enalapril	19.32 ± 3.19[b]	-40.11	70.15 ± 7.40[a]	34.94
Diabetic treated with Paricalcitol	17.87 ± 1.04[b]	-44.60	48.94 ± 0.63[b]	-5.99
Diabetic treated with Enalapril and Paricalcitol	20.46 ± 1.06[b]	-36.57	71.58 ± 3.47[a]	37.49
F-probability	P<0.001		P<0.001	
LSD at 5% level	6.230		12.609	
LSD at 1% level	8.429		17.059	

- Data are expressed as mean ± SE. Number of detected samples in each group is six.
- Means, which share the same superscript symbol(s) are not significantly different.
- Percentage changes were calculated by comparing diabetic control group with normal control group and diabetic treated groups with diabetic control group.
