# Supplementary material for: Effects of enalapril and paricalcitol treatment on diabetic nephropathy and renal expressions of TNF-α, p53, caspase-3 and Bcl-2 in STZ-induced diabetic rats
Source: PLoS One. 2019 Sep 17;14(9):e0214349. doi: 10.1371/journal.pone.0214349 (PMC6748411; doi:10.1371/journal.pone.0214349)
Supplement: S4 Table — (PDF) [file pone.0214349.s004.pdf]

Table 4: Effects of enalapril and paricalcitol on kidney GST and catalase activities in diabetic rats

	GST (U/g tissue)	% change	Catalase (U/g tissue)	% change
Normal	1350.00 ± 9.31[a]	-	6.83 ± 0.17[a]	-
Diabetic control	838.50 ± 8.65[c]	-37.88	3.71 ± 0.28[d]	-45.68
Diabetic treated with Enalapril	953.55 ± 4.03[bc]	13.72	5.22 ± 0.61[b]	40.70
Diabetic treated with Paricalcitol	1204.50 ± 8.89[a]	43.64	4.14 ± 0.23[cd]	11.59
Diabetic treated with Enalapril and Paricalcitol	1138.46 ± 9.27[ab]	35.77	5.07 ± 0.25[bc]	36.65
F-probability	P<0.01		P<0.001	
LSD at 5% level	241.36		1.014	
LSD at 1% level	326.54		1.371	

- Data are expressed as mean ± SE. Number of detected samples in each group is six.
- Means, which share the same superscript symbol(s) are not significantly different.
- Percentage changes were calculated by comparing diabetic control group with normal control group and diabetic treated groups with diabetic control group.
