# Supplementary material for: Effects of enalapril and paricalcitol treatment on diabetic nephropathy and renal expressions of TNF-α, p53, caspase-3 and Bcl-2 in STZ-induced diabetic rats
Source: PLoS One. 2019 Sep 17;14(9):e0214349. doi: 10.1371/journal.pone.0214349 (PMC6748411; doi:10.1371/journal.pone.0214349)
Supplement: S5 Table — (PDF) [file pone.0214349.s005.pdf]

Table 5: Effects of enalapril and paricalcitol on serum fasting and postprandial glucose levels in diabetic rats.

	Fasting serum glucose (mg/dl)	% change	Postprandial serum glucose (mg/dl)	% change
Normal	70.01 ± 5.91[d]	-	98.00 ± 5.34[d]	-
Diabetic control	229.40 ± 12.56[a]	231.26	255.75 ± 7.67[a]	160.96
Diabetic treated with Enalapril	135.24 ± 6.91[b]	-41.04	190.66 ± 16.10[b]	-25.45
Diabetic treated with Paricalcitol	103.60 ± 5.09[c]	-54.83	157.41 ± 11.93[c]	-38.45
Diabetic treated with Enalapril and Paricalcitol	83.6 ± 3.66[cd]	-63.55	101.33 ± 2.56[d]	-60.37
F-probability	P<0.001		P<0.001	
LSD at 5% level	21.806		29.009	
LSD at 1% level	29.502		39.247	

- Data are expressed as mean ± SE. Number of detected samples in each group is six.
- Means, which share the same superscript symbol(s) are not significantly different.
- Percentage changes were calculated by comparing diabetic control group with normal control group and diabetic treated groups with diabetic control group.
