# Supplementary material for: Effects of enalapril and paricalcitol treatment on diabetic nephropathy and renal expressions of TNF-α, p53, caspase-3 and Bcl-2 in STZ-induced diabetic rats
Source: PLoS One. 2019 Sep 17;14(9):e0214349. doi: 10.1371/journal.pone.0214349 (PMC6748411; doi:10.1371/journal.pone.0214349)
Supplement: S6 Table — (PDF) [file pone.0214349.s006.pdf]

Table 6: Effects of enalapril and paricalcitol on serum insulin and C-peptide levels in diabetic rats.

	**Insulin (ng/ml)**	**% change**	**C-peptide (ng/ml)**	**% change**
Normal	2.633 ± 0.111^{a}	-	4.733 ± 0.264^{a}	-
Diabetic control	0.606 ± 0.189^{c}	-76.98	0.774 ± 0.109^{c}	-83.64
Diabetic treated with Enalapril	1.833 ± 0.152^{b}	202.47	4.776 ± 0.097^{a}	517.05
Diabetic treated with Paricalcitol	1.413 ± 0.195^{b}	133.16	4.200 ± 0.146^{a}	442.63
Diabetic treated with Enalapril and Paricalcitol	1.640 ± 0.204^{b}	170.62	3.240 ± 0.427^{b}	317.70
F-probability	P<0.001		P<0.001	
LSD at 5% level	0.5063		0.7084	
LSD at 1% level	0.6850		0.9854	

- Data are expressed as mean ± SE. Number of detected samples in each group is six.
- Means, which share the same superscript symbol(s) are not significantly different.
- Percentage changes were calculated by comparing diabetic control group with normal control group and diabetic treated groups with diabetic control group.
