# Supplementary material for: Effects of enalapril and paricalcitol treatment on diabetic nephropathy and renal expressions of TNF-α, p53, caspase-3 and Bcl-2 in STZ-induced diabetic rats
Source: PLoS One. 2019 Sep 17;14(9):e0214349. doi: 10.1371/journal.pone.0214349 (PMC6748411; doi:10.1371/journal.pone.0214349)
Supplement: S7 Table — (PDF) [file pone.0214349.s007.pdf]

Table 7: Immunohistochemical staining integrated intensity (.10^6) for the expression of TNF-α, p53, caspase-3 and Bcl-2 in kidney of normal, diabetic control and diabetic groups treated with enalapril, paricalcitol and their mixture.

	**TNF-α**	**p53**	**Caspase-3**	**Bcl-2**
Normal	0.194±0.043^{b}	1.076±0.157^{c}	0.349±0.046^{c}	17.320±1.024^{b}
Diabetic control	5.848±0.659^{a}	29.628±4.688^{a}	10.190±2.241^{a}	2.303±0.214^{c}
Diabetic treated with Enalapril	0.370±0.143^{b}	1.107±0.335^{c}	0.660±0.209^{c}	15.396±1.499^{b}
Diabetic treated with Paricalcitol	1.040±0.342^{b}	10.151±1.581^{b}	5.704±0.733^{b}	12.421±4.078^{bc}
Diabetic treated with Enalapril and Paricalcitol	0.268±0.083^{b}	1.406±0.342^{c}	0.389±0.083^{c}	30.224±7.056^{a}
F-probability	P<0.001	P<0.001	P<0.001	P<0.01
LSD at 5% level	1.074	7.009	3.338	11.768
LSD at 1% level	1.528	9.969	4.748	16.739

- Data are expressed as mean ± SE. Number of replicates in each group is 3.
- The integrated intensities were calculated for positive immunohistochemical reactions in a standard measuring frame of the captured images,
- Means, which share the same superscript symbol(s) are not significantly different.
