# Supplementary material for: Effects of enalapril and paricalcitol treatment on diabetic nephropathy and renal expressions of TNF-α, p53, caspase-3 and Bcl-2 in STZ-induced diabetic rats
Source: PLoS One. 2019 Sep 17;14(9):e0214349. doi: 10.1371/journal.pone.0214349 (PMC6748411; doi:10.1371/journal.pone.0214349)
Supplement: S8 Table — (PDF) [file pone.0214349.s008.pdf]

Table 8: Stained area percent for the expression of TNF-α, p53, caspase-3 and Bcl-2 in kidney of normal, diabetic control and diabetic groups treated with enalapril, paricalcitol and their mixture.

	**TNF-α**	**p53**	**Caspase-3**	**Bcl-2**
Normal	0.096 ± 0.022^{b}	0.654 ± 0.140^{c}	0.342 ± 0.169^{c}	8.637 ± 0.511^{b}
Diabetic control	2.916 ± 0.328^{a}	14.774 ± 2.338^{a}	5.082 ± 1.118^{a}	1.148 ± 0.107^{c}
Diabetic treated with Enalapril	0.195 ± 0.070^{b}	0.553 ± 0.167^{c}	$0.\ 330 \pm 0.010^{c}$	7.678 ± 0.747^{b}
Diabetic treated with Paricalcitol	0.518 ± 0.170^{b}	5.062 ± 0.788^{b}	2.844 ± 0.365^{b}	8.448 ± 1.335^{b}
Diabetic treated with Enalapril and Paricalcitol	0.133 ± 0.413^{b}	0.701 ± 0.171^{c}	0.194 ± 0.042^{c}	15.121 ± 3.518^{a}
F-probability	$P<0.001$	$P<0.001$	$P<0.001$	$P<0.01$
LSD at 5% level	0.535	3.498	1.675	5.455
LSD at 1% level	0.761	4.976	2.382	7.759

- Data are expressed as mean ± SE. Number of replicates in each group is 3.
- Means, which share the same superscript symbol(s) are not significantly different.
